# From Smoking-Permitted to Smokefree Prisons: A 3-Year Evaluation of the Changes in Occupational Exposure to Second-Hand Smoke Across a National Prison System

**DOI:** 10.1093/annweh/wxaa073

**Published:** 2020-08-05

**Authors:** Evangelia Demou, Ruaraidh Dobson, Helen Sweeting, Ashley Brown, Scott Sidwell, Rachel O’Donnell, Kate Hunt, Sean Semple

**Affiliations:** 1 MRC/CSO Social and Public Health Sciences Unit, Institute of Health and Wellbeing, University of Glasgow, Glasgow, UK; 2 Institute for Social Marketing and Health, University of Stirling, Stirling, UK; 3 Scottish Prison Service, Edinburgh, UK

**Keywords:** PM_2.5_, prisons, second-hand smoke exposure, smokefree policy, smoking, TIPs, Tobacco in Prisons study, workplace

## Abstract

**Objectives:**

Prisons in Scotland were one of the few workplaces exempt from the 2006 comprehensive smoking ban in indoor public places, excluding the prison workforce from the health benefits of smokefree workplaces. The November 2018 introduction of comprehensive restrictions on smoking in Scottish prisons aimed to protect prison staff and people in custody from the harmful impacts of second-hand smoke (SHS) exposure. This study presents SHS exposure data gathered after smokefree policy implementation and compares these with data gathered during and before policy development.

**Methods:**

Dylos DC1700 monitors were used to measure concentrations of fine particulate matter (PM_2.5_) derived from SHS across Scotland’s 15 prisons. Six days of fixed-site monitoring (09.00 22 May 2019 to 09.00 28 May 2019) were conducted in residential halls in each prison 6 months post-smokefree policy implementation. Prison staff task-based measurements were conducted to assess concentrations of SHS in various locations (e.g. gyms and workshops) and during specific activities (e.g. cell searches, maintenance, and meal service). Utilizing the fixed-site monitoring data, typical daily PM_2.5_ exposure profiles were constructed for the prison service and time-weighted average (TWA) exposure concentrations were estimated for the typical shift patterns of residential staff pre- and post-smokefree policy implementation. Staff perceptions of changes to SHS exposure were assessed using online surveys.

**Results:**

Analysis of both fixed-site and mobile task-based PM_2.5_ measurements showed the smokefree policy implementation was successful in reducing SHS exposures across the Scottish prison estate. Measured PM_2.5_ in residential halls declined markedly; median fixed-site concentrations reduced by more than 91% compared with measures in 2016 before policy announcement. The changes in the TWA concentrations across shifts (over 90% decrease across all shifts) and task-based measurements (89% average decrease for high-exposure tasks) provide evidence that prison staff exposure to SHS has significantly reduced. Following smokefree policy implementation, the majority of staff reported no longer being exposed to SHS at work.

**Conclusions:**

To our knowledge, this is the first comprehensive international study to objectively measure SHS levels before, during, and after implementation of a smokefree policy across a country’s prison system. The findings confirm that such a policy change can be successfully implemented to eliminate occupational exposures to SHS. The results are highly relevant for other jurisdictions considering changes to prison smoking legislation.

## Introduction

In 2006, Scotland became one of the first countries in the world to introduce comprehensive legislation on smokefree indoor public places (Scottish Parliament, 2005), to protect workers and the public from exposure to second-hand smoke (SHS). Evaluation of this law demonstrated that SHS concentrations in pubs and bars, key targets of the legislation, fell by 86% following implementation (Ayres *et al.*, 2009). Reduced exposure to SHS led to a 17% reduction in hospitalization for acute coronary syndrome in the year after introduction (Pell *et al.*, 2008) and an annual decline of 18% in children’s admission to hospital for asthma over the 3 years from 2006 to 2009 (Mackay *et al.*, 2010).

However, certain workplaces were not covered by the legislation, on the basis that they were also ‘homes’, preventing some workers from receiving the health benefits of a smokefree workplace. Notably, Scotland’s prisons continued to permit people in custody to smoke tobacco in their room (cell) and some outdoor areas, while staff were not allowed to smoke within prison premises or grounds. The three-phase, multi-methods Tobacco in Prisons (TIPs) study adopted a natural experimental methodology to assess the process and impact of the introduction of a smokefree prisons policy on 30th November 2018. Phase 1 (2016–July 2017) occurred before the policy was formulated, Phase 2 (July 2017–November 2018) in anticipation, and Phase 3 (December 2018–May 2020) after its introduction (Hunt *et al.*, 2017). TIPs included measurements of SHS exposure within prisons on three occasions, in: Phase 1 (2016); the week of implementation (2018); and 6 months after implementation (2019) (Hunt *et al.*, 2017). Previous studies have examined changes in SHS within several prisons as part of national or state-wide policy changes (Proescholdbell *et al.*, 2008; Thornley *et al.*, 2013), or where only one or some prisons are becoming smokefree (Jayes *et al.*, 2019). The TIPs project is uniquely comprehensive in its focus on the entire Scottish prison estate, with research conducted in all prisons in the country. In 2019, these prisons housed over 8000 people in custody and employed over 4000 staff. The Scottish Prison Service (SPS) has 13 publicly managed prisons and two by private sector operators under contract to SPS (SPS, 2020). The majority of people in custody are men (94%) and young and white (94%). One site houses young males aged 16–21; women (who form a minority ~5% of people in custody in Scotland, as elsewhere) are housed in four sites including in one female-only prison. Prisons often house a mix of populations in respect of sex, remanded or convicted status and sentence length; 14 of the 15 are ‘closed’ establishments. Those housed within the only ‘open’ prison can work within the community and are permitted some home leave prior to release. There is a wide mixture of building age, types, and design, with considerable variability in ventilation systems, across and within the 15 sites. The oldest prison building was first used in 1863 while the newest was opened in 2012. Building capacity ranges from just over 100 to 1300.

Phase 1 TIPs research conducted in 2016 demonstrated high levels of SHS in Scottish prisons, particularly in residential halls (Semple *et al.*, 2017) and partially informed the decision to introduce the smokefree policy across Scotland’s 15 prisons (SPS, 2017). Air quality monitoring in the week of the implementation of the smokefree policy (November 2018) demonstrated that SHS levels in prisons fell dramatically compared with 2016 (Semple *et al.*, 2020). The sites and methods used to undertake fixed measurements of fine particulate matter (PM_2.5_) in the air in residential halls in 2016 and 2018 were the same and the 2018 figures provided a snapshot immediately after the new policy had been introduced. However, it was not feasible in 2018 to also conduct mobile, task-based measures as had been done in 2016, and the 2018 figures only provided a snapshot immediately after the new policy had been introduced. It was considered worthwhile to repeat the measures after the policy had time to embed fully. For example, any illicit trading of tobacco within the prisons could affect tobacco availability, and so also SHS levels. Therefore, as part of Phase 3 of the TIPs study, a further round of air quality monitoring was conducted in May 2019, 6 months after smokefree implementation, to assess levels of SHS within Scotland’s prisons and to estimate prison staff personal exposures in comparison with 2016 data (Semple *et al.*, 2017).

This is the first study to measure change in SHS across a whole national prison estate over the period when a prison system has gone entirely smokefree. We aim to determine the success of this policy in reducing SHS concentrations in all of Scotland’s prisons by analysing results from measurements taken in 2016 (pre-ban) and 2018 (as the ban was being introduced) and comparing them with new measurements made 6 months after the smokefree policy came into force.

## Methods

### Ethics

Ethical approval for TIPs was granted by both the Scottish Prison Service (SPS) Research Access and Ethics Committee and the University of Glasgow’s College of Social Sciences Ethics Committee (reference numbers: 400150213 and 400150214).

### Air quality monitoring

As in 2016 (Semple *et al.*, 2017) and 2018 (Semple *et al.*, 2020), 15 Dylos DC1700 monitors were used to detect fine PM_2.5_ in the air within prisons. Monitors of this kind have been used previously in a range of studies to detect PM_2.5_ derived from SHS (Semple *et al.*, 2015). Monitors were calibrated individually against a TSI SidePak instrument set to a previously accepted value for SHS-PM_2.5_, and calibration factors applied to the estimated PM_2.5_ mass concentration determined by the device. Each prison was assigned the same monitor used in the 2018 phase of air quality measurements to minimize the effect of sensor drift. It was not possible to use the same monitors as had been used during the 2016 measurement period.

### Fixed-site area monitoring

Six days of fixed-site area monitoring were conducted in each of Scotland’s 15 prisons between ~09.00 22 May 2019 and ~09.00 28 May 2019. A Dylos monitor was placed in a residential hall or landing area in each prison as close as possible to the locations used in 2016 and 2018 by members of prison staff (trained in protocols for the measurements). At the end of the fixed-site monitoring period, members of the research team visited prisons to download data from the monitor and discuss protocols for the task-based monitoring with prison staff.

### Mobile task-based monitoring

Prison staff were asked to use the Dylos DC1700 monitor while completing between four and eight tasks in different areas, to assess SHS concentrations in various locations (such as gyms and workshops) and during different work-based activities (e.g. cell searches, maintenance, and meal service). Although the chosen tasks were left to the discretion of prison staff in each prison, based on their knowledge and perceptions of areas with potential SHS exposures, staff were asked to include, where possible, the same monitoring locations/activities undertaken in 2016 (Semple *et al.*, 2017). The monitoring period for each ‘task-based’ measurement lasted for around 30 min.

### Outdoor air pollution

As outdoor air pollution can be a confounding factor for indoor air monitoring, hourly outdoor PM_2.5_ concentration data were downloaded from www.scottishairquality.co.uk for the monitoring period. As in previous analyses, data from the closest available reference PM_2.5_ monitor to each prison were used (Semple *et al.*, 2017, 2019).

### Statistical analysis

Mean values of residential hall fixed-site monitoring results were taken for each prison over the full 6-day period. To test the significance of change between 2016 and 2019, mean PM_2.5_ concentrations were log-transformed and a single paired *t*-test conducted across mean concentrations from all prisons.

Since SHS levels varied according to time of day, time-specificity of exposure for residential staff was conducted by utilizing the continuous fixed-site measurements of PM_2.5_ across all prisons as an indicator of SHS exposure levels to obtain a more detailed account of exposures of residential staff during different shift patterns (Jenkins *et al.*, 1996; Klepeis *et al.*, 1996; Jaakkola and Jaakkola, 1997). Time-weighted average (TWA) shift exposures were estimated for a ‘typical’ residential officer in a Scottish prison, using information on shifts provided by the SPS. TWA exposures show a worker’s daily exposure to a pollutant (typically normalized to an 8-h day or to duration of a shift), taking into account both the average levels of exposure in an area and the time spent in a particular area. All fixed-site measurements, across the 15 prisons, were combined and daily exposure profiles were calculated. Using the combined daily SHS exposure profile estimate across the prison service, average, minimum, and maximum TWA exposures for four typical shifts were estimated. The four shifts were: ‘early shift’ (modelled as a 6 h shift; staff on this shift have responsibility of unlocking cells first thing in the morning); a ‘day shift’ (modelled as an 8 h shift); a ‘back shift’ (modelled as a 9 h shift); and the ‘night shift’ (modelled as a 10 h shift).

Statistical analysis was conducted in Microsoft Excel, R version 3.6.1 and IBM SPSS Statistics v23.

### Survey questionnaire

Online surveys were circulated to staff in each TIPs phase in: November–December 2016 (Phase 1); May–July 2018 (Phase 2); and May–July 2019 (Phase 3) (Sweeting *et al.*, 2019, 2020). Emails including the survey link and information on the TIPs study were prepared by the research team and circulated to contacts in each prison to forward to all prison staff; one, two, and three reminder emails were circulated in Phases 1, 2, and 3, respectively. These online surveys included questions on opinions on smoking bans and e-cigarettes in prisons, smoking behaviour, health, employment, and socio-demographic characteristics and on perceived exposure to SHS, enabling comparison of staff self-perceived personal SHS exposure across the three phases of the study. Participation was not mandatory and no incentives were offered to prison staff. The survey took approximately 10–15 min to complete.

## Results

### Data integrity

Integrity of the residential hall fixed-site 6-day monitoring data was generally high. A total of 126 777 min of data were recorded. One prison (#11) was missing just over 1 day of data in three blocks (with 7163 min, just under 5 days, recorded), while another (#8) was missing a single block of 18 h of data (7536 min, i.e. 5 days and 6 h, recorded).

Integrity of the task-based measurements was also high. Mobile task-based measurements were returned from 14 of the 15 prisons, covering a total of 3073 min of data. In total, 77 different task-based measurements were completed (range = 3–7 different measures, lasting 10–165 min). Each measurement was assigned a code based on the location where the measurement was taken or the activity being undertaken at the time of measurement. Codes were consistent with those used in the 2016 measurements (Semple *et al.*, 2017).

### Fixed-site area monitoring

PM_2.5_ levels in prison halls declined substantially in every prison between 2016 (before policy announcement) and 2019 (Table 1) (*P* < 0.001 overall). Median PM_2.5_ concentrations of the 6-day fixed-site measurements, over this time period, decreased by more than 91% compared with 2016 concentrations.

**Table 1.** Residential hall fixed-site PM_2.5_ monitoring results from 2016 and 2019 monitoring rounds.

Prison ID	2016		2019	
	Indoor mean PM_2.5_, µg m^−3^ (standard deviation)	Outdoor^a^ mean PM_2.5_, µg m^−3^	Indoor mean PM_2.5_, µg m^−3^ (standard deviation)	Outdoor^a^ mean PM_2.5_, µg m^−3^
1	11.2 (9.4)	6.6	1.7 (1)	3.5
2	54.6 (37.5)	11.4	3.3 (2.4)	3.5
3	28.8 (16.7)	10.5	3.6 (1.8)	2.9
4	135.9 (189.4)	5.2	5.9 (15.6)	3.0
5	48.6 (62.4)	9.4	3.4 (2)	3.8
6	28.5 (15.8)	5.9	2.8 (2)	4.0
7	36 (15.1)	6.4	2.3 (1.7)	2.4
8	31.7 (16.2)	22.8	2.1 (6.7)	4.5
9	23.4 (13.5)	5.3	2.1 (1.5)	3.1
10	49.2 (48.6)	5.7	6.8 (4.9)	2.8
11	32 (20.8)	11.5	3.6 (4.4)	4.3
12	19.8 (12.1)	12.6	1.5 (0.9)	3.5
13	35.3 (21)	5.3	3 (4)	2.9
14	31.1 (18.8)	6.5	3.3 (2.7)	2.4
15	10.5 (8.7)	7.7	1.7 (2.4)	3.5
Median	31.7	6.6	3.0	3.5

^*a*^Outdoor mean PM_2.5_ concentrations obtained from: www.scottishairquality.co.uk

Median indoor PM_2.5_ concentrations were close to five times greater than outdoor PM_2.5_ concentrations in 2016 but were less than outdoor concentrations by 2019 (Table 1). This provides clear evidence of the presence of a significant indoor source of PM_2.5_ (SHS) in 2016, which was no longer detectable in 2019.

**Figure 1.**
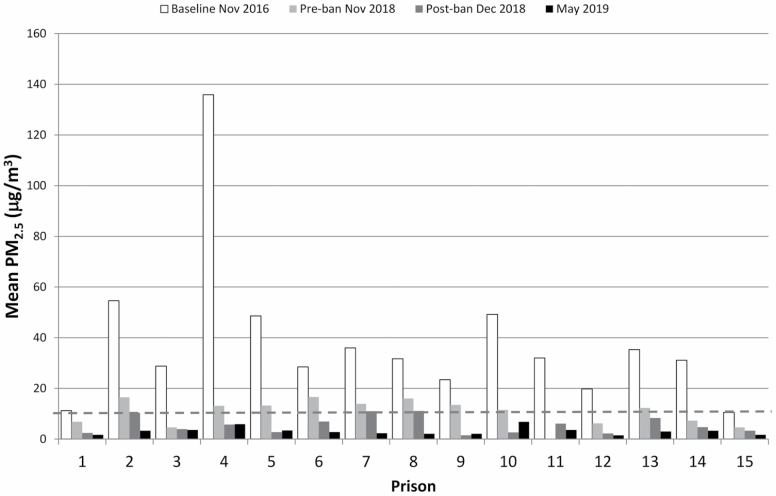
Fixed-site area monitoring results from 2016, 2018 (pre- and post-ban), and 2019. Detected PM_2.5_ levels declined at each measuring point.

PM_2.5_ concentrations measured in 2019 were lower than those post-ban in 2018, but the difference was not statistically significant. Comparative results from 2016, 2018 (1 day pre-ban), 2018 (1 day post-ban), and 2019 area monitoring are given in Fig. 1, showing the large scale of the decline.

### Task-based monitoring

**Figure 2.**
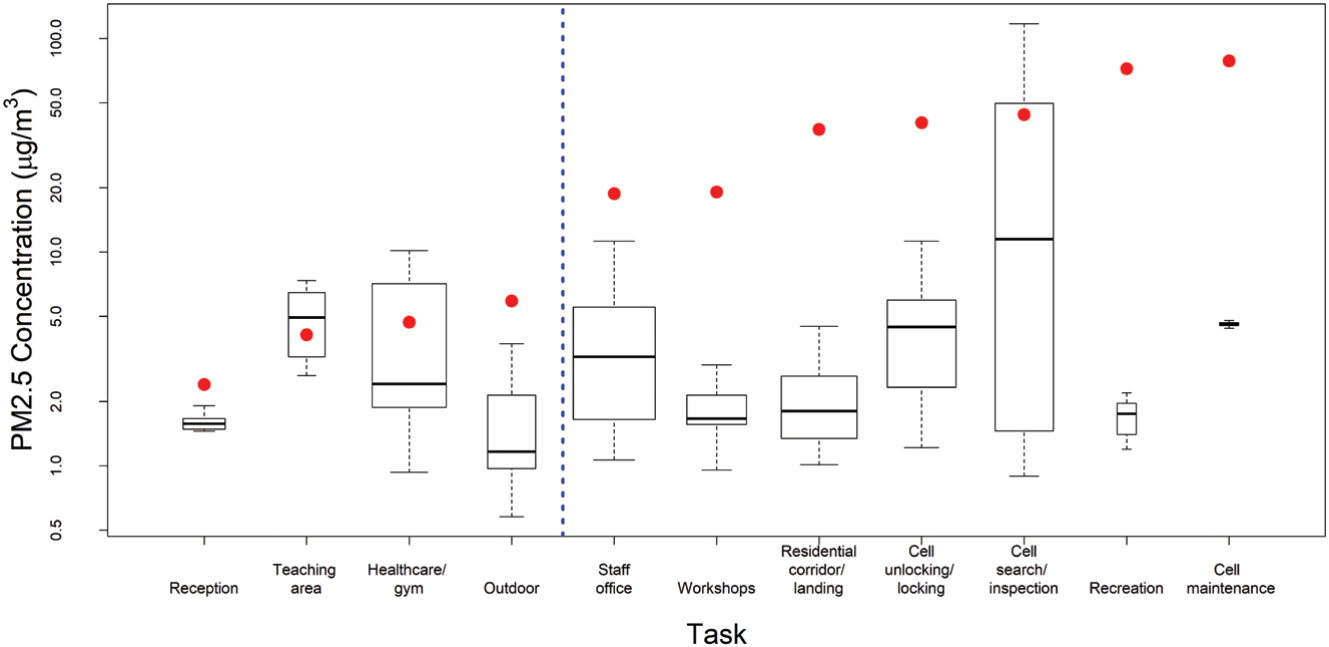
Task-based PM_2.5_ concentrations (log scale) combined across all 15 prisons and categorized by location/activity. Red point indicates median PM_2.5_ concentration of same location/activity type from 2016 measurements.

Fig. 2 shows boxplots of the results of the task-based measurements post-ban (2019), with the median value of the 2016 pre-announcement measurements matched to the same location/activity types (Semple *et al.*, 2017). Post-ban, all task-based measurements were low; the only task with a median >10 µg m^−3^ was cell searches. Apart from teaching areas (which already had some of the lowest levels in 2016), all locations/tasks saw a significant reduction in PM_2.5_ concentrations. Overall there was an average reduction of 70% in PM_2.5_ concentrations for the task-based measures. For the locations/activities that were identified in our 2016 measurements as most likely *not* to have significant SHS exposures (i.e. reception, teaching areas, healthcare/gym, and outdoor), concentrations remained low and decreased in most areas. For the locations/activities where concentrations were considered in 2016 most likely to lead to considerable exposure to SHS among residential staff, such as unlocking/locking cells and cell searches (see measures to the right-hand side of the vertical dotted line in Fig. 2), PM_2.5_ concentrations decreased on average by 89%, with a maximum change in median concentrations of 98% for recreational areas and a minimum of 74% in cell searches. Morning cell unlocking, which was identified as an exposure ‘hot spot’ in our Phase 1 (2016) measurements, saw a reduction in the median PM_2.5_ concentration of 89%.

### TWA exposures by shift

**Figure 3.**
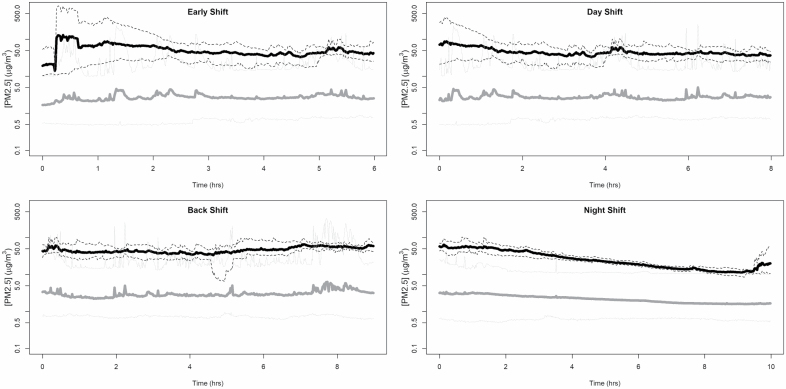
PM_2.5_ exposure concentrations (average, min, and max) by shift pre (black line) and post (grey line) implementation of the smokefree policy in prisons.

Our 2016 air quality measurements prior to the smokefree policy indicated that the staff group most at risk of being exposed to SHS was operational/residential staff working in the residential halls (Semple *et al.*, 2017). Residential staff effectively spend the majority of their shift in the residential halls, apart from their break. The residential hall 6-day fixed-site and task-based measurements shown above (Figs 1 and 2) evidence a significant change in workplace exposures to PM_2.5_ as a marker for SHS; these indicate that most shift TWA exposures to SHS are likely to be extremely low post-ban. Typically, residential shift staff work 1 week on early shifts (lasting around 6 h) and then rotate to a longer shift the following week. Early shift residential staff are responsible for cell unlocking, and throughout the day, staff carry out cell inspections (both identified as high-exposure tasks in 2016). Fig. 3 and Supplementary Fig. S1, available at *Annals of Work Exposures and Health* online demonstrate a typical 24-h average exposure profile across the prison service, over the four main shifts. These profiles are based on all daily residential hall fixed-site measurements combined across the prisons before (2016) and 6 months after (2019) implementation of the smokefree policy.

The average exposure daily profile clearly shows the effect of the smokefree policy in the exposure patterns throughout a typical working day. Before the policy was put in place (2016), high peak exposures were recorded first thing in the morning (Semple *et al.*, 2017; Fig. 1), when cell unlocking occurred (Fig. 3 and Supplementary Fig. S1, available at *Annals of Work Exposures and Health* online), whilst PM_2.5_ concentrations were low at night. Post-ban, the average exposure profile (Fig. 3 and Supplementary Fig. S1, available at *Annals of Work Exposures and Health* online) shows little diurnal variation, with the average concentration being below those observed during an average night shift in 2016, before the introduction of the smokefree policy.

Specifically, the exposure profiles for residential staff on the four shifts that occur in a typical day, showed reductions in TWA exposures of over 90% across all shifts (Table 2). TWA shift exposures reduced the most (by 95%) for residential staff working on an early shift. The 2019 exposure profiles also demonstrate that, with the implementation of the smoking ban, there is very little difference in TWA exposures between the different shift groups. Also, in 2019, 6 months post-ban, all TWA exposures per shift were low and lower than outdoor PM_2.5_ concentrations (Table 1).

**Table 2.** TWA shift exposures pre- and post-smoking ban across all prisons.

	2016			2019			Pre–post reduction
	TWA_average_- [PM_2.5_] (µg m^−3^)	TWA_min_-[PM_2.5_] (µg m^−3^)	TWA_max_-[PM_2.5_] (µg m^−3^)	TWA_average_- [PM_2.5_] (µg m^−3^)	TWA_min_-[PM_2.5_] (µg m^−3^)	TWA_max_-[PM_2.5_] (µg m^−3^)	Δ-TWA_average_ [PM_2.5_]
Early shift (6 h)	53.55	23.31	150.27	2.74	0.62	33.15	94.9%
Day shift (8 h)	46.10	25.82	87.96	2.79	0.67	31.09	93.9%
Back shift (9 h)	45.87	31.91	67.04	3.06	0.73	39.57	93.3%
Night shift (10 h)	28.33	21.90	37.24	2.21	0.61	12.56	92.2%

### Staff perceptions of exposure to SHS at work

The online staff survey in Phase 3 had the lowest return rate for staff from all phases, at 16%, compared with return rates of 27 and 31% for Phases 1 and 2, respectively (Sweeting *et al.*, 2020). The majority of staff who responded to the survey in Phase 3, worked full-time (average working week: 36.4 h week^−1^; range: 10–55 h week^−1^). Throughout the TIPs study and prior to implementation of the smokefree policy, only people in custody were allowed to smoke within prisons and on prison grounds. The proportions of staff reporting *no* exposure to other people’s cigarette smoke at work rose from 19% (Phase 1), to 27% (Phase 2), to 74% in Phase 3. For the operational staff, i.e. those who are most likely to be working in residential halls, the proportion reporting *no* exposure to other people’s cigarette smoke at work rose from 13% in Phase 1 to 72% in Phase 3. The proportion of operational staff reporting 11 or more hours’ exposure a week dropped from 54 to 11% between Phases 1 and 3.

## Discussion

### Summary of main findings

Implementation of the smokefree policy was successful, with no significant incidents reported, across the Scottish prison system (SPS, 2019). The results of residential hall fixed-site monitoring show that PM_2.5_ concentrations declined substantially in all prisons between 2016 (pre-ban) and 2019 (6 months post-ban).

Both fixed-site monitoring and task-based measurements showed large reductions in SHS-related PM_2.5_ and suggest that staff and people in custody now experience daily exposures to PM_2.5_ concentrations that are similar to those found in most smokefree environments. The 2019 fixed-site monitoring results are comparable to, or lower than, outdoor PM_2.5_ on the same days, demonstrating that no substantial indoor emissions of PM_2.5_ were occurring in prisons during those measurements. The task measurements indicate that the high pre-ban exposures to SHS associated with particular staff groups and tasks (e.g. cell searches) (in 2016) have been significantly reduced, protecting staff from its health harms and substantially reducing the differences in exposure between different groups of staff working within the prison.

### Results in context of previous literature

These final TIPs (Phase 3) SHS exposure measures show the large decline in measured PM_2.5_ during the week of policy implementation (Semple *et al.*, 2020) was maintained 6 months later, with fine particulate emissions generated from smoking activity reduced to close to zero.

TIPs is unique as it represents the first research to observe changes in objectively measured SHS across an entire national prison system during the development and introduction of a smokefree policy, incorporating three time points (before any policy change prohibiting smoking had been formulated, in the week of implementation of the ban, and 6 months later when the ban had had time to ‘bed in’). The large (91%) median reduction in PM_2.5_ 6 months post-ban compared with levels in Phase 1 (before the policy was formulated) is comparable to studies conducted around the introduction of smokefree public places legislation in Scotland in 2006, when levels of measured PM_2.5_ in bars declined by 86% (Semple *et al.*, 2007). However, it is larger than reductions observed in other studies that have measured PM_2.5_ levels in samples of prisons before and after smokefree policy implementation, including: four English prisons as they went smokefree in 2016 (66%) (Jayes *et al.*, 2019); six North Carolinian prisons in 2005–2006 (77%) (Proescholdbell *et al.*, 2008); and one New Zealand prison in 2010–2011 (57%) (Thornley *et al.*, 2013). All 15 Scottish prisons became smokefree on the same day; by contrast, other jurisdictions have introduced smokefree prison policies piecemeal or over a longer implementation period (Ritter *et al.*, 2012). English prisons, for example, became smokefree in stages between 2016 and 2018 (ASH, 2018). The larger median decline in Scottish prisons (91%), compared with that measured in a sample of English prisons (66%) (Jayes *et al.*, 2019) may be attributable to increased awareness of the policy among people in custody and staff in Scotland due to widespread internal communications and media attention, or may have been related to other factors (such as the forms of support available within prisons to support people in custody in abstaining from smoking following policy implementation, including sale of rechargeable e-cigarettes).

Several organizational measures, including a sufficient time (16 months) between policy change announcement and smoking ban implementation, a strong communication strategy, changes in the provision of smoking cessation services, and the availability of e-cigarettes (rechargeable vaping devices) for people in custody (PHS, 2018), could have impacted on the successful implementation of the Scottish prison smokefree policy and good compliance with smokefree rules, as confirmed by levels of PM_2.5_ measured in 2019. These explanations are explored in more detail elsewhere, using data from qualitative interviews with staff and people in custody conducted in Phase 3 of TIPs; these data suggest that both staff and people in custody reported that the transition had been less troublesome than they had expected. The change from smoking-permitted to smokefree was also associated with, amongst staff, an increase in support for smoking bans in prisons and a decrease in concerns over implementation challenges and risks (Sweeting *et al.*, 2020). The availability of e-cigarettes in Scottish prisons may have been one factor to positively impact the immediate success of the implementation of the policy (Brown *et al.*, 2019a, 2020; Sweeting *et al.*, 2019). Rechargeable e-cigarettes were made available in Scottish prisons shortly before smokefree policies were implemented and given free to people in custody who smoked in Scottish prisons for a limited time in an attempt to facilitate management of the change for smokers. In pre-implementation (Phase 1) TIPs research, people in custody reported that they would be more likely to support smokefree policies if e-cigarettes were permitted (Brown *et al.*, 2019b). Recent findings on the experiences of and attitudes to use of e-cigarettes by people in custody are reported elsewhere (Brown *et al.*, 2020).

### Strengths and limitations

A strength of this study is that we were able to undertake objective measures of SHS exposures across an entire national prison system before, during and after implementation of smokefree policy. Prisons are unique workplaces with numerous organizational complexities and challenges. Working independently but in close partnership with the SPS, we were able to train selected members of prison staff to measure air quality in both fixed-site positions to a standardized protocol, over a 6-day period and during specific tasks and in non-residential locations that were considered potential ‘hot spots’ of exposure before implementation of the ban. In addition, our comprehensive measurements across all prisons enable estimation of the range of possible exposures based on different prison types and roles.

We note three limitations to the study. Firstly, personal SHS sampling was not possible due to the operational and resource constraints and challenges associated with the roles of prison staff, and especially residential prison staff. Therefore, modelling personal exposures assume residential staff are exposed to the average concentration of a residential hall throughout their shift and does not account for specific tasks and individual movements. Secondly, the low staff response rate to the survey is a further limitation, and staff perceptions of their personal exposures reported here may not be representative of all Scottish prison staff. Other internet-based surveys with occupational cohorts have also reported low response rates (Delclos *et al.*, 2005; Dykema *et al.*, 2013). Contrary to the Phase 3 (post-implementation) objective SHS measures, some staff reported being exposed to SHS, with a small proportion reporting exposure for 11 or more hours a week. One possible explanation could be that staff are actually reporting exposure to ‘vaping smoke’ rather than ‘tobacco smoke’. Although we tried to make this distinction clear in our survey, we cannot discount this as a reason for the reporting of SHS exposure. Despite this, comparisons showed very marked drops in self-report SHS exposures between Phases 1 and 3. Thirdly, as in previous analyses (Semple *et al.*, 2017, 2020) our comparison of indoor PM_2.5_ concentrations with concurrent data gathered for outdoor air used the nearest available local authority monitoring station. The distance of these stations from each prison was variable. The median distance was 16 km, but one prison was 133 km from the nearest available PM_2.5_ monitoring device in the 2016 data collection period. Given the low outdoor PM_2.5_ concentrations measured at all three time points [median values across the outdoor sites of 6.6 µg m^−3^ (2016), 5.0 µg m^−3^ (2018), and 3.5 µg m^−3^ (May 2019)], it is unlikely that the distance between prison and monitoring station has introduced substantial bias.

We further note that the Dylos monitors could have detected PM_2.5_ associated with vaping during the 2018 and 2019 monitoring periods, potentially confounding our results. While there have been no studies on the use of Dylos DC1700 monitors to detect second-hand e-cigarette aerosol (SHA), similar optical particle counters have been used to do so (Tzortzi *et al.*, 2020). SHA is detectable but dissipates quickly (likely due to evaporation of the propylene glycol medium), unlike SHS which can persist in the indoor environment for several hours (Semple and Latif, 2014). For this reason we would not expect SHA to have a significant effect on measured PM_2.5_ concentrations.

## Conclusions

This study is the first to assess SHS exposure in a prison system throughout a process of organizational change (the introduction of comprehensive smokefree policy) prior to formulating that policy change, in the period between announcement of the policy and its implementation, and after the new policy became part of the organization’s status quo. Working collaboratively with the SPS, learning from the organization and its staff, and accounting for the organizational challenges and opportunities of the prison system, we were able to objectively assess changes in SHS exposure for prison staff and people in custody. The results, which represent one distinct element of the multi-phase, multi-method TIPs study, showed the implementation of the smokefree policy was successful in effectively eliminating SHS exposure for both staff and people in custody across the Scottish prison system. PM_2.5_ fixed-site concentrations substantially reduced across all prisons between the 2016 (pre-ban) and 2019 (post-ban) periods; post-ban, they are now similar to those found in smokefree home environments. Furthermore, task-based measures and exposure models demonstrated that no extra SHS exposure is experienced by any categories of staff or on particular shifts. These results, along with our further TIPs findings on the perceptions and experiences of staff and people in custody relating to the smokefree policy across the prison service, and forthcoming modelling of outcomes, are highly relevant for other prison services considering organizational and policy changes relating to indoor smoking rules.

## Supplementary Material

wxaa073_suppl_supplementary-Material
