# Supplementary material for: From Smoking-Permitted to Smokefree Prisons: A 3-Year Evaluation of the Changes in Occupational Exposure to Second-Hand Smoke Across a National Prison System
Source: Ann Work Expo Health. 2020 Aug 5;64(9):959–69. doi: 10.1093/annweh/wxaa073 (PMC7668237; doi:10.1093/annweh/wxaa073)

**SUPPLEMENTARY INFORMATION**

Figure S1. Average, minimum and maximum daily PM_2.5_ exposure profiles pre (top) and post (bottom) ban.


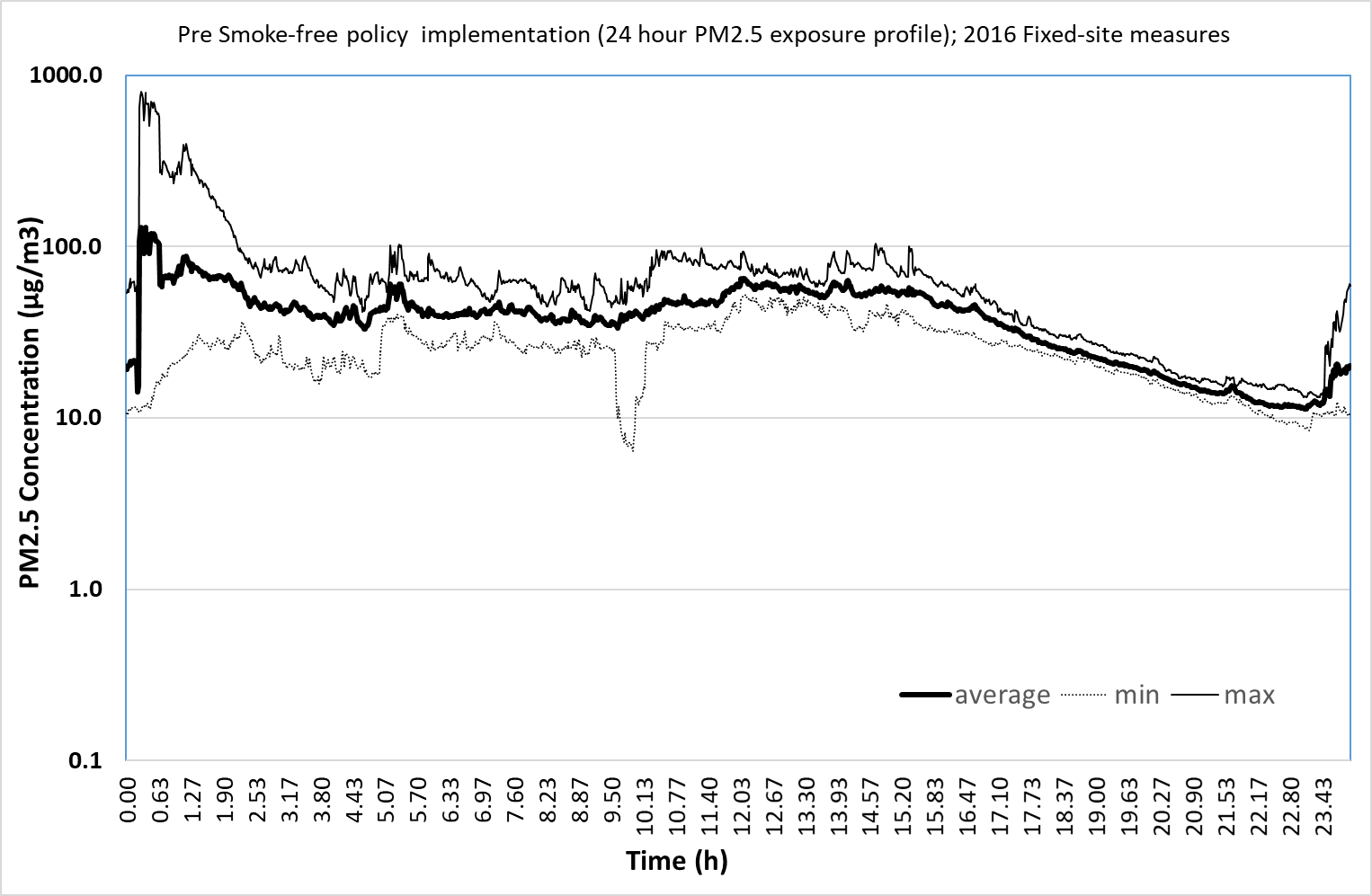


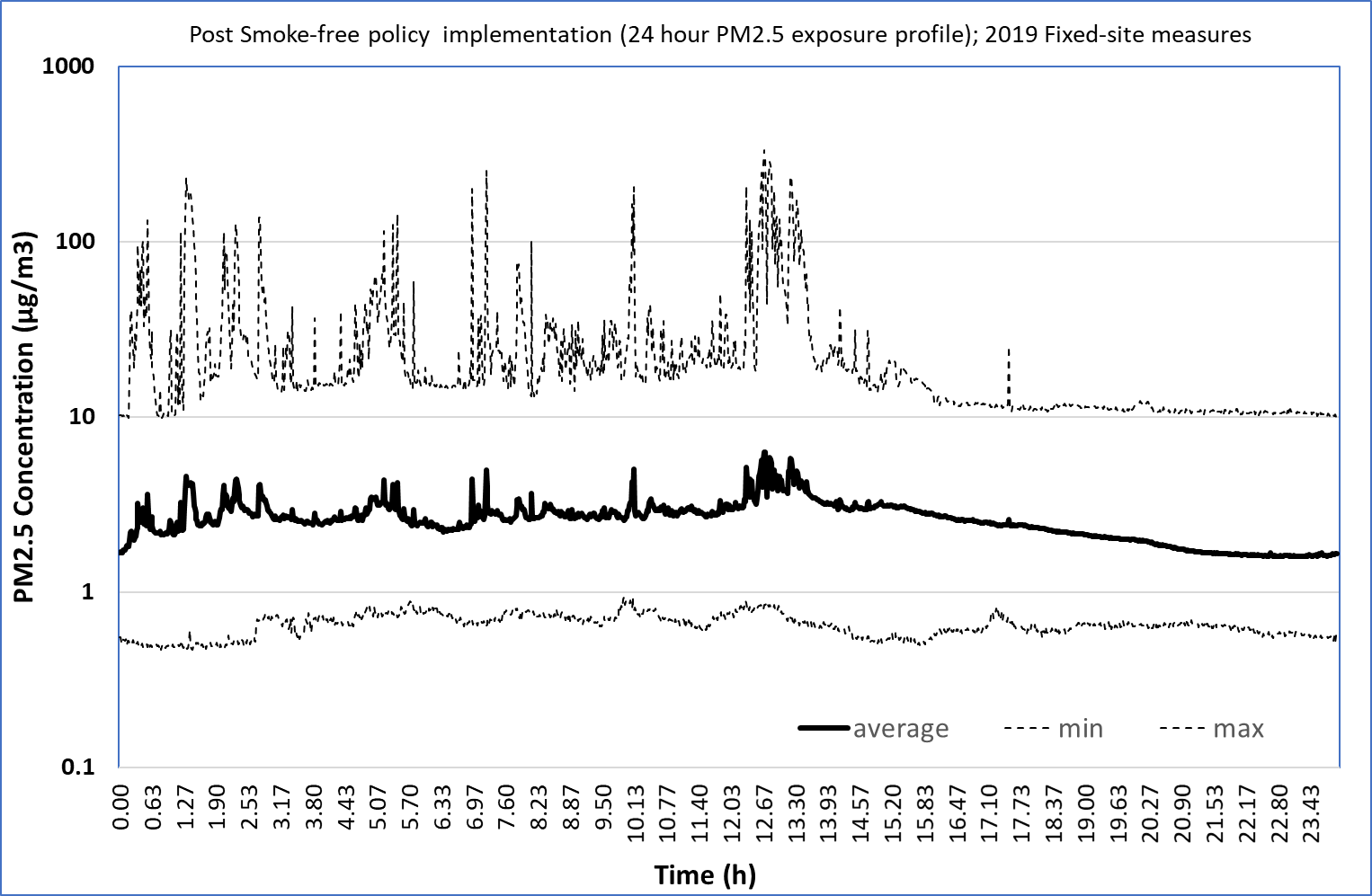

Supplement: wxaa073_suppl_supplementary-Material [file wxaa073_suppl_supplementary-material.docx]
